# The Aperta type of neural tube defect: The relevant experience in a local community with the diversity of the presentation

**DOI:** 10.25122/jml-2023-0190

**Published:** 2023-10

**Authors:** Wissam Saleh Hakim, Ali Saleh Aljanabi

**Affiliations:** 1 Department of Surgery, College of Medicine, University of Al-Qadisiyah, Iraq

**Keywords:** Neural Tube Defects (NTDs), spina bifida, anencephaly

## Abstract

A series of birth defects known as neural tube defects (NTDs) appear when the neural tube fails to fully or partially close during fetal development. In nations without folic acid supplementation, their incidence ranges from 0.5 to 2 per 1,000 births. The purpose of our study is to estimate the prevalence of NTDs and define the workup for newborn infants with an open neural tube in Al-Qadissiyah, Iraq. This 18-year descriptive retrospective analysis included all babies with NTD diagnoses at the Maternity and Child Teaching Hospital in Al-Qadissiyah Governorate, Iraq. Over the research period, 187 cases of NTDs were evaluated. NTDs presented a male predominance and an incidence rate of 9.4 per 1,000 births, with spina bifida (67.9%), encephalocele (24.02%), and anencephaly (8.02%) being the most prevalent defects. The typical gestational age was 36±5, whereas the typical maternal age was 30±5. It should be noted that 29.9% of women did not take folic acid supplements during the first trimester, while one-third of moms did not benefit from medical supervision during pregnancy. In 65.2% of instances, an antenatal diagnosis was made, and cesarean section was the mode of delivery in 87,8% of cases. Other than that, 64.7% of women lived in low socioeconomic conditions, and 67.9% were from rural areas. The relevance of reinforcing and maximizing folic acid measures throughout the periconceptional phase is emphasized by the fact that NTDs require high intensity and advanced care.

## INTRODUCTION

Spina bifida and anencephaly are the two most prevalent severe birth defects of the central nervous system, also known as neural tube defects (NTDs). They develop when the neural tube fails to close fully during embryonic development [1].

NTDs are the major acause of death in the first year of life due to their severity. Moreover, the level of care for NTDs differs considerably among countries. Over 75% of infants with NTDs died due to anencephaly, which is always lethal and causes death before age five [2].

NTDs prevalence varies significantly between regions and nations, ranging from 1 to 10 per 1,000 births [3]. However, estimates of the prevalence of birth defects and the associated disabilities might differ. Moreover, they are frequently influenced by the nature of the disorders and the ways they are categorized [4].

The nutritional status of mothers may be a moderator of NTD risks as per experimental and clinical findings [5]. Folate molecules participate in one-carbon metabolism to provide purines and thymidine for the synthesis of deoxyribonucleic acid (DNA) and s-adenosyl methionine (SAM). SAM is the universal methyl group donor needed for many methylation reactions [6].

In advanced countries, medical and surgical management has evolved to the point where the majority of newborns with spina bifida will live to adulthood [7]; however, they will experience long-term disability and treatment expenditures, which continue to be significant barriers. Complex deformities known as NTDs have multiple etiological causes (e.g., nutritional, environmental, and genetic) [8].

## MATERIAL AND METHODS

### Study design

This study evaluated all occurrences of NTDs, whether they were present alone or in combination with other anomalies, at the Maternity and Children Teaching Hospital in the Al-Qadisiyah governorate from 2003 to 2022. In this investigation, irrespective of the term or outcome of the pregnancy, we included all women whose fetuses or newborns presented a single or combined NTD.

### Data collection

Data included maternal demographics and reproductive characteristics (maternal age, socioeconomic level, folic acid supplementation, mode of delivery, and living area), infant features (gestational age, birth weight, sex, NTD type, and whether isolated or associated anomaly). Apart from that, all births were examined carefully for birth defects. A doctor confirmed birth defects in infants and photos were taken with informed consent.

## RESULTS

A total of 187 cases were examined in this investigation. The median maternal age was <35 years in 123 cases (65.7%), and >35 years in 64 cases (34.3%). One-third of mothers gained no noticeable benefits from the monitoring of prenatal care. In contrast, 131 mothers (70.1%) did not take supplements containing folic acid during the first trimester of pregnancy. In addition, 121 mothers (64.7%) lived in low socioeconomic conditions, eight of them (4.3%) had a good socioeconomic status, while 58 (31%) were considered middle class.

The results revealed that 60 mothers (32.1%) lived in an urban area, while 127 (67.9%) lived in a rural area. Otherwise, with just two instances of opting for a therapeutic pregnancy interruption, the antenatal diagnosis was carried out in 122 (65.2%) of the cases. The majority of deliveries were live births, and most deliveries (164 cases) were done by cesarean section (87.8%), with vaginal deliveries being encountered in only 23 cases (12.2%).

NTDs were present alongside other malformations in 45 cases (24.1%) and isolated in 142 cases (75.9%). Two infants had a history of encephalocele and spina bifida. Out of the analyzed cases, 9.6% of babies were delivered prematurely (<37 weeks; n=18), whereas 12.8% of infants had low birth weights (<2,500 g; n=24), and spina bifida (n=127; 67.9%), the most typical form of NTD. Approximately 80% of infants having spina bifida were born alive, with the majority of affected births having a lesion in the lumbosacral (n=116; 62.3%) or lumbar (n=56; 29.9%) regions of the spine (Table 1).

**Table 1:** Neural Tube Defects (NTDs) Sites

Sites of NTDs	n	%
**Lumbosacral**	116	62.03
**Lumbar**	56	29.9
**Thoracic**	5	2.6
**Cervical**	5	2.6
**Occipital**	5	2.6

**Figure 1:**
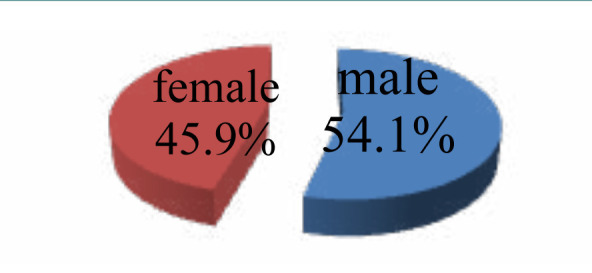
Sex Distribution of NTD

Female infants were less likely than male ones to have spina bifida (male: n=101 male, 54.1%; female: n=86, 45.9%), while anencephaly was more common among female infants (Figure 1).

The second-most prevalent NTD identified was anencephaly (n=15; 8.02%). Although approximately half of the cases were live births, all fetuses passed away very quickly. The third most frequent NTD was encephalocele (n=45; 24.06%). The majority of newborns having encephalocele were born alive, and the encephalocele was mostly located in the occipital region (n=5; 2.6%).

Furthermore, meningocele discovered in 122 cases (65.9%) was the most common NTD, followed by myelomeningocele, found in 46 cases (24.5%). On the other hand, the lipomeningocele was presented just in 13 cases (6.9%), with a similar occurrence of lipomyelomeningocele and myelocystocele in three cases only (1.6%) (see Table 2).

**Table 2:** Neural Tube Defects (NTDs) Types

Types of NTDs	n	%
**Meningocele**	122	65.9
**Meningomyelocele**	46	24.5
**Lipo Meningocele**	13	6.9
**Lipo Meningomyelocele**	3	1.6
**Myelocystocele**	3	1.6

## DISCUSSION

Through folic acid supplementation, antenatal ultrasound diagnosis, prenatal modification of maternal serum alpha-fetoprotein, and occasional pregnancy termination, the incidence of NTDs is declining in all industrialized and developing nations.

Nevertheless, in most developing nations, including Iraq, NTD incidence is still high. In this study, the incidence rate was (9.4/1,000), which is high compared to the previous research in the same governorate (8.5/1,000) [6]. It is higher in Najaf (27/1,000 live births) [11] and Fallujah (95/1,000 live births) [12], but lower in Basra (4.3/1,000 live births) [9] and Baghdad (4.4/1,000 live births) [10] governorates. Table 3 shows our overall incidence of anencephaly and spina bifida in several governorates in Iraq.

**Table 3:** Incidence of neural tube defects (NTDs) in different governorates of Iraq

Governorate	All NTDs	Spina bifida	Anencephaly	Encephalocele
Al-Anbar^14^	3.3	2.2	0.9	0.2
Diwaniah^6^	8.4	3.7	2.3	2.4
Basrah^9^	4.34	1.13	1.74	-
Najaf^11^	27.0	9.0	10.4	7.0

* Superscript number indicates the number of NTDs cases in the Iraqi governorate

The variations in incidence could result from genetic, socioeconomic, nutritional, and distribution factors, in addition to the influence of data collection type and the diagnostic criteria. Other than that, the consequences of exposure to environmental factors, such as depleted uranium, poor maternal nutrition, folate deficiency, lack of antenatal programs, low dietary folate supplementation, and psychosocial stress, are believed to be responsible for the rise in the incidence of NTDs in Iraq.

In our NTDs instances, we noticed a male predominance. Among 187 cases, 101 (54.1%) were male and 86 (45.9%) were female.

This result supports another study done in Kampala, Uganda, which showed NTDs were less common among females compared to males [13]. Subsequently, other evidence suggests that a higher risk of NTDs may be related to gender, with women presenting a higher prevalence [14, 15]. Additional literature research demonstrated a significant frequency of NTDs in females, especially in cases of anencephaly [16]. We also notice that men are more likely to have spina bifida than women [17]. Anencephaly is the most prevalent NTD in Western nations, with a rate of one birth out of every 10,000 in the United States (US) [18-19].

This was in contrast to our findings, which demonstrated that spina bifida was far more common than anencephalic NTD anomalies, with an incidence rate of spina bifida (67.9%), encephalocele (24.02%), and anencephaly (8.02%). Hence, this is in agreement with a similar study [14], which observed the incidence was higher for spina bifida, followed by encephalocele and anencephaly.

The lumbosacral (62.03%) and dorsolumbar (62.03%) areas were the most commonly encountered NTD sites in our research. Meanwhile, the thoracic and cervical regions were the least common sites with equal distribution (2.6%). This is consistent with the North American pattern that illustrated a higher predominance of the lumbosacral site [20] but was in contrast to the Western pattern for the distribution. The thoracolumbar was the most common NTD site [21], with hydrocephaly being associated with 60 (32.08%) of our NTD patients.

It has been observed that patients with thoracic spina bifida and the majority of those with lumbosacral spina bifida are more likely to develop hydrocephaly [22]. In addition to anencephaly and encephalocele, cases of spina bifida will have about an 80-85% probability of having underlying hydrocephalus anomalies [23]. In our research, the prevalence of NTDs was 127 cases (67.9%) in rural areas. In comparison, 60 cases (32.1%) came from urban areas, contrasting the findings of Linda Barlow-Mosha *et al*. [13], which showcase that 85% of the women with NTDs were referred from urban and periurban areas rather than rural areas.

Our results found that 122 cases (65.2%) were diagnosed antenatally. In only 56 cases (29.9%) folic acid was supplemented before birth, and, as the mode of delivery, cesarean section was used in 164 cases (87.8%). This contrasted the findings of Khenata Forci *et al*. [24], a study that mentioned that among women, one-fourth did not benefit from prenatal care monitoring. During the first trimester of pregnancy, 59% of women did not use folic acid supplements, and none consumed B9 vitamins during the periconceptional phase. Only seven cases of NTDs resulted in cesarean delivery, but just four cases (9.1%) made the prenatal diagnosis, which was performed in 63% of cases and led to a therapeutic pregnancy interruption.

Nonetheless, this research revealed a high prevalence of pregnancies in women under the age of 35 (65.7%), with the remaining 34.3% of cases occurring in women over 35, excluding all instances of abortion. However, this contradicted the findings of Ahmad Behrooz *et al*. (2007) [7]. Out of 56 women whose pregnancies were impacted by NTDs, 12.5% were aged 10 to 20; 39 (69.7%) were aged 21 to 30; 10 (17.8%) were aged 31 to 40 [25].

### Study limitations

Only infants who were born alive were included in this retrospective analysis. Due to lethal syndromes and diseases linked to NTDs (e.g., Meckel-Gruber syndrome), there are considerable numbers of stillbirths and terminations of pregnancies that are not included in statistics. Subsequently, this study covers NTD in one medical center. A long-term follow-up is required to detect all abnormalities, including functional abnormalities and disabilities, which were not assessed in the current study.

## CONCLUSION

Rural locations with inadequate antenatal care lead to an increased incidence of affection of the fetus. We believe folate supplements are necessary to stop the recurrence of NTDs in infants of high-risk mothers. To determine the causal influence and the link between maternal and paternal factors, dietary factors, and NTDs, more research with a large sample size is required.

## Special thanks

I would like to thank Dr. Mohammed J. Aboud, the expert who participated in the survey validation for this study. The survey validation could not have been carried out successfully without his enthusiastic participation and input.
